# Hypermutable Non-Synonymous Sites Are under Stronger Negative Selection

**DOI:** 10.1371/journal.pgen.1000281

**Published:** 2008-11-28

**Authors:** Steffen Schmidt, Anna Gerasimova, Fyodor A. Kondrashov, Ivan A. Adzuhbei, Alexey S. Kondrashov, Shamil Sunyaev

**Affiliations:** 1 Division of Genetics, Brigham and Women's Hospital, Harvard Medical School, Boston, Massachusetts, United States of America; 2 Department of Biochemistry, Max Planck Institute for Developmental Biology, Tübingen, Germany; 3 Life Sciences Institute, University of Michigan, Ann Arbor, Michigan, United States of America; 4 Department of Ecology and Evolutionary Biology, University of Michigan, Ann Arbor, Michigan, United States of America; 5 Section on Ecology, Behavior, and Evolution, Division of Biological Sciences, University of California San Diego, La Jolla, California, United States of America; University of Aarhus, Denmark

## Abstract

Mutation rate varies greatly between nucleotide sites of the human genome and depends both on the global genomic location and the local sequence context of a site. In particular, CpG context elevates the mutation rate by an order of magnitude. Mutations also vary widely in their effect on the molecular function, phenotype, and fitness. Independence of the probability of occurrence of a new mutation's effect has been a fundamental premise in genetics. However, highly mutable contexts may be preserved by negative selection at important sites but destroyed by mutation at sites under no selection. Thus, there may be a positive correlation between the rate of mutations at a nucleotide site and the magnitude of their effect on fitness. We studied the impact of CpG context on the rate of human–chimpanzee divergence and on intrahuman nucleotide diversity at non-synonymous coding sites. We compared nucleotides that occupy identical positions within codons of identical amino acids and only differ by being within versus outside CpG context. Nucleotides within CpG context are under a stronger negative selection, as revealed by their lower, proportionally to the mutation rate, rate of evolution and nucleotide diversity. In particular, the probability of fixation of a non-synonymous transition at a CpG site is two times lower than at a CpG site. Thus, sites with different mutation rates are not necessarily selectively equivalent. This suggests that the mutation rate may complement sequence conservation as a characteristic predictive of functional importance of nucleotide sites.

## Author Summary

Mutations occur in some sites in the genome more frequently than in others. Similarly, mutations in some sites have greater consequences than in others. The effect of mutations might not be independent of the frequency with which mutations occur. Indeed, sites where mutations happen frequently will be preserved if the effects of these mutations are severe or will otherwise be allowed to mutate if there are no consequences for the organism. We compared both human–chimpanzee differences and sequence variation among humans in protein coding genes. We found that highly mutable nucleotide sites, such as the dinucleotide CpG, are on average more important and more frequently preserved by natural selection. Using this information, together with other features such as sequence conservation, opens a new perspective to predict the effect of human mutations, including their potential involvement in diseases.

## Introduction

The functional and phenotypic effects of mutations and, consequently, the strength of negative selection vary widely among nucleotide sites in any genome. At the opposite ends of the continuum, mutations at some sites are effectively neutral, while mutations at some other sites are lethal. Nucleotide sites can be subdivided, according to their molecular function, into classes with different typical strengths of negative selection. Generally, rapidly evolving segments of intergenic regions and introns, as well as most of synonymous coding sites, are controlled by only weak selection or even by no selection at all. Slowly evolving segments of intergenic regions and introns, as well as UTRs and non-synonymous coding sites, are under much stronger selection (e.g., [1]–[8]). However, even within such functional classes, the strength of negative selection varies widely among individual sites (e.g., [9]–[12]).

The rate of spontaneous mutation is also not uniform across individual sites [13]–[15]. The standard deviation of the mutation rate at a site may be comparable to its mean. Moreover, some rare hot-spot sites may mutate much more frequently than an average site. Thus, the mutation rate at a site depends both on its local sequence context (e.g., [16]–[19]) and on its global location within the genome [13]–[15], although these dependencies are rather different in different groups of organisms [19],[20]. In particular, in mammals the 5′CpG3′ context substantially increases the rate of transversions, and especially transitions [16]–[19],[21].

Mutation and selection are generally thought to be independent evolutionary forces [22]. In other words, the rate with which a mutation occurs is routinely assumed to be independent of the effect of this mutation on fitness. Inferences of the strength of selection on specific genes and sites within genes usually rely on this assumption. Although selection for reduced mutability is stronger at sites where mutations are more deleterious [23], it is hard to imagine adaptive fine-tuning of mutation rates at the level of individual nucleotide sites. Thus, one might expect selective constraint and mutability to vary more or less independently across individual sites.

However, another phenomenon may lead to a seemingly counterintuitive association between stronger negative selection and higher mutation rates. Sites that are under weak or no selection are free to evolve and to get rid of hypermutable contexts. In contrast, negative selection will preserve such contexts at functionally important sites, provided that they confer a higher fitness. In particular, non-synonymous [24] and even synonymous [21],[25] coding sites of mammalian genomes are enriched, relative to what is expected at a neutral mutational equilibrium, by CpG contexts, leading to a substantially higher mutation rate within coding exons than within introns.

Here we consider human non-synonymous coding sites and subdivide them into just two classes – those within and those outside CpG contexts, because in mammals this context exerts by far the strongest influence on the mutation rate [19]. Then, we compare the rates of human-chimpanzee divergence [26] and the levels of intrahuman polymorphism at coding sites that are within *vs.* outside CpG context. We have found that the strength of negative selection acting at non-synonymous coding sites is substantially higher within hypermutable CpG contexts.

## Results

If identical nucleotides at identical sites within codons of identical amino acids are under the same selection, regardless on whether they are located within or outside CpG context, then this context would equally impact the mutation rate, the rate of divergence between species, and the level of intraspecies nucleotide diversity. If, however, negative selection is stronger within CpG context, this context would elevate the level of nucleotide diversity and especially the rate of divergence, to a lesser extent than the mutation rate.

### Impact of CpG Context on Mutation Rates

It is well known that in mammals CpG context substantially increases the mutation rate; however, the exact magnitude of this effect has not been established with certainty. We used three sources of information on the impact of CpG context on the rates of transitions and transversions: 1) direct data on Mendelian diseases in humans [18], 2) Bayesian Markov Chain Monte Carlo analysis of evolution of several species of mammals [19], and 3) parsimony-based analysis of human-chimpanzee-orangutan genome alignments (Table 1). The third analysis must underestimate the impact of CpG context on transversion and especially transition rates, because two nucleotide substitutions, one on the edge leading to a sister species (human or chimpanzee) and the other on the edge leading to the outgroup (orangutan), can happen within a CpG context. Such occurrences will lead to underestimation of the fraction of sites that were within CpG context in the common ancestor of human and chimpanzee and, thus, of the fraction of allele substitutions that destroy a CpG context. Indeed, this underestimation is evident from Table 1. Thus, below we will use the mean values of the first two estimates and will assume that in humans CpG context increases the rate of transitions by the factor of 14.5, and the rate of transversions by the factor of 3.5.

**Table 1:** Estimates of the impact of CpG context on the mutation rates of transitions and transversions.

*Ratio*	Kondrashov (2003)	Hwang & Green (2004)	*average*	(human-chimp)-orangutan
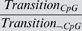	15.4	13.7	14.5	12.2
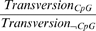	2.8	4.2	3.5	2.4

The last column contains ratios computed using a ((human-chimp)-orangutan) alignment.

### Impact of CpG Context on the Rate of Evolution and Intraspecies Diversity at Non-Synonymous Sites

We used human-chimpanzee-orangutan alignments of coding sequences to compare the rates of a particular nucleotide substitution that causes a particular amino acid replacement within *vs.* outside CpG context (CpG vs. ⌝CpG). For example, a P→L replacement, caused by a C→T transition, can occur within (C**C**G→C**T**G; the site of substitution is boldfaced) or outside (e.g., C**C**C→C**T**C) CpG context. The common ancestor of humans and chimpanzees, as revealed by the orangutan outgroup, carried, at all the loci we studied, Target_P→L CpG_ = 18,088 of CCG codons, and Target_P→L⌝CpG_ = 185,826 of CCA, CCT, or CCC codons (Table 2). There were 215 and 284 P→L replacements (Replacements_P→L CpG_ and Replacements_P→L⌝CpG_), caused by C→T transitions, within CpG and outside CpG contexts, respectively. Thus the impact of CpG context on the rate of P→L replacements in the course of human-chimpanzee divergence is
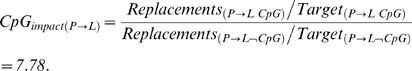
(1)


**Table 2:** Non-synonymous substitutions in human-chimp divergence and human polymorphism data.

		Divergence										Diversity		
		Macaque as outgroup					Orangutan as outgroup							
		CpG	⌝CpG	CpG_target_	⌝CpG_target_	CpG_impact_	CpG	⌝CpG	CpG_target_	⌝CpG_target_	CpG_impact_	CpG	⌝CpG	CpG_impact_ (orangutan)
**Transitions**	V→I	368	481	11,714	128,771	*8.41*	318	353	10,814	103,463	*8.62*	247	190	*12.44*
	V→M	197	217	14,951	92,303	*5.60*	176	154	13,593	76,037	*6.39*	166	88	*10.55*
	A→T	352	582	26,266	269,079	*6.20*	345	408	22,865	212,630	*7.86*	213	237	*8.36*
	G→S	177	238	14,793	118,403	*5.95*	150	154	12,891	93,855	*7.09*	130	83	*11.40*
	G→R	124	205	12,561	131,213	*6.32*	96	155	10,913	101,692	*5.77*	93	70	*12.38*
	D→N	119	236	16,226	191,210	*5.94*	119	160	14,172	150,842	*7.92*	104	99	*11.18*
	E→K	150	307	21,964	286,222	*6.37*	106	229	19,512	223,672	*5.31*	121	132	*10.51*
	S→L	162	79	14,345	56,932	*8.14*	122	62	12,596	43,892	*6.86*	101	29	*12.14*
	P→L	323	401	20,988	229,606	*8.81*	215	284	18,088	185,826	*7.78*	179	137	*13.42*
	A→V	275	477	22,769	260,480	*6.60*	235	363	19,841	215,997	*7.05*	176	198	*9.68*
**Transversions**	V→L	26	151	26,938	227,412	*1.45*	43	161	39,449	284,400	*1.93*	27	85	*2.29*
	V→F	9	41	9,937	97,770	*2.16*	7	22	9,088	78,368	*2.74*	8	16	*4.31*
	A→P	24	120	25,938	268,617	*2.07*	22	89	22,542	212,311	*2.33*	12	58	*1.95*
	A→S	69	242	25,983	268,739	*2.95*	69	171	22,589	212,393	*3.79*	26	87	*2.81*
	G→R	37	104	27,601	253,250	*3.26*	18	31	23,805	196,963	*4.80*	12	31	*3.20*
	G→C	19	39	14,635	118,204	*3.93*	14	20	12,755	93,721	*5.14*	5	14	*2.62*
	G→W	1	17	8,088	54,094	*0.39*	4	10	7,229	43,326	*2.40*	4	5	*4.79*
	D→H	11	91	16,118	191,065	*1.43*	9	65	14062	150,747	*1.48*	6	39	*1.65*
	D→Y	8	54	16,115	191,028	*1.76*	8	36	14,061	150,718	*2.38*	4	21	*2.04*
	E→Q	32	203	21,846	286,118	*2.06*	24	149	19,430	223,592	*1.85*	13	74	*2.02*
	P→Q	27	37	20,692	74,730	*2.64*	24	23	17,897	59,091	*3.45*	11	15	*2.42*
	T→K	22	43	17,764	66,600	*1.92*	11	30	15,900	53,435	*1.23*	10	13	*2.59*
	A→E	30	50	22,524	70,891	*1.89*	28	30	19,634	56,882	*2.70*	12	13	*2.67*
	P→R	18	120	20,683	229,325	*1.66*	13	100	17,886	185,642	*1.35*	15	51	*3.05*
	A→G	25	144	22,519	260,147	*2.01*	20	114	19,626	215,748	*1.93*	11	79	*1.53*
	T→R	16	50	17,758	66,607	*1.20*	10	34	19,416	223,477	*3.39*	6	11	*6.28*
	F→L	13	68	6,884	77,474	*2.15*	7	47	6,173	62,281	*1.50*	3	19	*1.59*
	C→W	6	13	5,004	47,722	*4.40*	2	7	4,293	37,600	*2.50*	0	4	*0.00*
	H→Q	15	82	6,737	55,640	*1.51*	17	46	5,903	43,819	*2.74*	5	25	*1.48*
	N→K	9	95	7,605	71,708	*0.89*	13	68	6,812	57,756	*1.62*	5	39	*1.09*
	S→R	25	74	8,433	71,141	*2.85*	20	49	7,227	56,443	*3.19*	4	35	*0.89*
	D→E	31	145	10,551	90,245	*1.83*	14	104	9,121	72,379	*1.07*	11	40	*2.18*

Column heads: identity of an amino acid change; the data for transitions is shown in the upper part of the table, transversions below. Columns: divergence data with macaque or orangutan as outgroup followed by diversity data computed from human non-synonymous SNP. CpG/⌝CpG: number of changes within/outside CpG context; CpG_target_/⌝CpG_target_: number of targets within/outside CpG context. CpG_impact_ impact of CpGs as calculated in formula 1. The nsSNP data shown here is based on the Applera data set using human-chimp alignments to determine the direction of the mutation.

This analysis relies on the identification of the human-chimpanzee ancestral state using orangutan as outgroup. To test whether possible erroneous identifications affect our results, we repeated the same analysis using the macaque outgroup, which must lead to more errors, because macaque is about three times more distant from the human-chimpanzee last common ancestor than orangutan. Also, all the analyses were performed only for human and chimpanzee coding sequences, under the assumption that the proportion of CpG context within these sequences is at equilibrium. Estimates of the impact of CpG context on the rates of evolution obtained in this way were only slightly higher than estimates obtained using the orangutan outgroup (data not reported).

**Figure 1:**
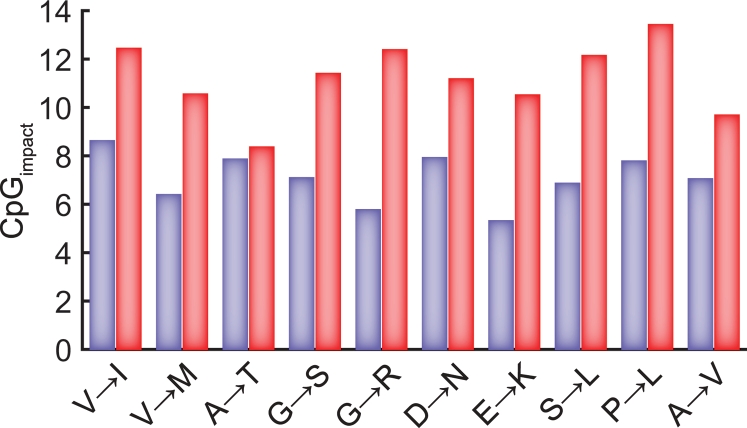
CpG impact on transitions in amino acid changes. The effect on human-chimpanzee divergence is shown in blue; the effect on non-synonymous SNPs in human in red.

For intraspecies nucleotide diversity, the number of SNPs that involve a particular amino acid change within and outside CpG context were used in equation (1), instead of the corresponding numbers of substitutions (Table 2). The direction of an amino acid change associated with a particular SNP was determined by the orthologous chimpanzee sequence. We assumed that the ratio of CpG vs. ⌝CpG target sizes for a particular amino acid replacement was the same as for human-chimpanzee divergence. Indeed, the SNPs we used were obtained by resequencing of ∼11,000 human loci [27] so that we can expect the nucleotide composition of this sample to be close to that of all protein-coding loci. The data on the impacts of CpG context on human-chimpanzee divergence and on intrahuman diversity are shown in Table 2 and in Figure 1. Thus, the impact of CpG context on the rate of divergence, i.e. the average ratio of the rates of divergence within vs. outsides CpG contexts, was 7.1 for transitions and 2.5 for transversions. The average ratio of values of intrahuman diversities for non-synonymous SNPs within vs. outsides CpG contexts was 11.2 for transitions and 2.4 for transversions (Table 3). If macaque instead of orangutan is used as an outgroup, the observed impacts of CpG context on the rates of divergence decline only slightly (6.8 instead of 7.1 for transitions, and 2.1 instead of 2.5 for transversions).

**Table 3:** Average impacts of CpG context for different types of sites using orangutan as outgroup.

CpG_impact_	Type	Divergence	Diversity
non-synonymous	transition	7.1	11.2
	transversion	2.5	2.4
synonymous	transition	8.6	11.7
	transversion	2.1	2.3
non-coding	transition	12.2	13.7
	transversion	2.4	2.0

We applied several tests to evaluate the significance of the difference of the impact of CpG context on non-synonymous divergence and diversity. This difference is insignificant for transversions and highly significant for transitions, according to the *χ^2^* test (*p* = 2.8·10^−16^). However, the *χ^2^* test does not stratify data according to amino acid replacements, which is essential in our case. We used two approaches to perform stratified analysis of contingency tables. First, we combined *p*-values of separate tests for each amino acid replacement, using Stouffer (*p*<2.2·10^−16^) and Fisher (*p* = 2.7·10^−16^) methods. We also applied Cochran-Mantel-Haenszel test, the standard test for stratified analysis of contingency tables (*p* = 4.6·10^−16^).

### Impacts of CpG Context at Synonymous and Non-Coding Sites

We measured the impacts of CpG context on rates of evolution and nucleotide diversity at synonymous coding and at non-coding sites (Table 3). As it was the case for non-synonymous sites, we assumed parsimony. Thus, the data on rates of evolution at non-coding sites shown in Table 3 are taken from ((human-chimpanzee)-orangutan) comparison shown in Table 1.

We can see that the impacts of CpG context on non-coding human-chimpanzee divergence and intrahuman nucleotide diversity are rather close to the corresponding impacts on the mutation rate, which is consistent with effective neutrality of most of the non-coding DNA in humans. The figures in Table 3 are likely to be slightly underestimated, due to substitutions in the outgroup lineage.

In contrast to non-coding sites, at synonymous sites the impacts of CpG context on human-chimpanzee divergence and intrahuman nucleotide diversity due to transitions, but not to transversions, are substantially lower than the corresponding impacts on the mutation rates, although still higher than the corresponding impacts at non-synonymous sites. This implies that some selection acts on synonymous transitions within CpG context, and that this selection is weaker than the corresponding selection at non-synonymous sites. Several analyses revealed weak selection favoring Cs and Gs at synonymous sites [25],[28].

## Discussion

Our results show that negative selection is stronger within CpG contexts than in less mutable sites at identical codon positions. We can see that the per nucleotide site rate of transitions, accepted in the course of human-chimpanzee divergence, is on average 7.1 times higher within CpG contexts than outside CpG contexts (Table 3). A comparison of this figure with the impact of CpG on the corresponding mutation rate (Table 1) suggest that a transition that occurred within CpG context gets fixed in the course of human-chimpanzee divergence with a probability of 7.1/14.5 = 0.49 of the probability of fixation of a transition that occurred outside CpG context. Thus, nucleotides within CpG context are protected by a stronger selection.

In the case of SNPs, we observed a similar but weaker effect. On average, non-synonymous SNPs caused by transitions are 11.2 times more common within CpG context than outside of it. Thus, a non-synonymous transition mutation that occurred within CpG context is observed as a SNP with a chance that constitutes only 11.2/14.5 = 0.77 of the chance of observing a transition that caused the same amino acid replacement but occurred outside CpG context.

In other words, in the case of transitions, CpG context increases the level of intrahuman diversity and in particular the rate of non-synonymous divergence less than proportionally to its impact on the mutation rate. This demonstrates that negative selection at non-synonymous sites within CpG context is stronger than at sites outside it. This seemingly counterintuitive pattern probably has a simple evolutionary explanation: nucleotide sites that are not under strong negative selection will eventually lose most of their hypermutable CpG contexts. Thus, hypermutable contexts must be disproportionally common at sites under strong negative selection.

It is not surprising that a stronger negative selection within CpG contexts affects the rates of evolution more than it affects intraspecies diversity. Indeed, a substantial fraction of SNPs that segregate within a population are nevertheless subject to negative selection that is strong enough to prevent their fixation [22]. The large difference between the impacts of CpG context on polymorphism and divergence suggests that the observed effect is mostly due to nucleotide sites under weak selection, which affects divergence more than polymorphism. Such sites are abundant in human protein coding genes [9]–[11],[29].

Predictably, the impacts of CpG context at mostly selectively neutral noncoding sites do not differ substantially from its impacts on the mutation rate. In contrast, coding synonymous sites within CpG contexts evolve slower and are less diverse within humans than what would be expected on the basis of the mutation rates alone. This is not surprising because the impact of CpG context must be sensitive to even weak selection [25],[28]. Indeed, CpG contexts are greatly underrepresented at purely neutral sites, but even a rather weak selection is expected to increase their prevalence substantially, as long as the coefficient of selection is of the order of the reciprocal of the effective population size or higher [22]. CpG contexts are much more common within synonymous sites than within non-coding sites [25].

CpG context exerts a much weaker influence on the rate of transversions than on the rate of transitions (see Table 1). Thus, it is not surprising that the effects, which we can easily observe in the case of transitions, are not visible in the case of transversions. More data are needed to determine if these effects, however weak, are still present in the case of transversions.

Our estimates of the impact of CpG context on divergence (Tables 2 and 3) are probably too low due to substitutions in the outgroup lineage. However, these estimates depend only slightly on whether orangutan or macaque is used as an outgroup, although in the second case the prevalence of multiple substitutions at a site should be much higher. Also, the estimates computed from only human and chimpanzee genomes assuming equilibrium of the CpG content are only slightly higher than the estimate obtained using an outgroup. Further, the estimate of the impact of CpG context on human-chimpanzee divergence due to transitions at non-synonymous sites is much lower than the corresponding estimate for non-coding sites computed using the same outgroup (Table 3). This indicates that the low impact of CpG contexts not just an artifact of the assumption of parsimony. Even under the impossible assumption that every site that is located within CpG context in either human or chimpanzee sequence was also located within CpG context in their last common ancestor, the resulting estimate of the impact of this context on the rate of divergence equals 12 and is still lower than CpG impact on raw mutation rate.

The analysis of intrahuman diversity relies on the chimpanzee sequence for determining the identity of ancestral alleles. Misidentification of ancestral alleles would result in an underestimation of the impact of CpG context because ancestral CpGs would preferentially evolve in the chimpanzee lineage. To evaluate a possible extent of this bias we repeated the analysis using major and minor alleles instead of inferred ancestral and derived alleles. The resulting estimate of the impact of CpG context on non-synonymous transitions is 11.5, which is only slightly higher than 11.2 (Table 2).

Negative selection can also be detected in polymorphism data independently of intraspecies nucleotide diversity through changes in the distribution of allele frequencies, because such selection causes an excess of low-frequency alleles. In particular, minor allele frequencies of non-synonymous SNPs that affect slowly evolving (conserved) protein sites are reduced [30],[31]. The excess of rare alleles was not statistically significant in the two datasets of human SNPs used in this study. The effect of weak negative selection on allele frequency distribution is expected to be much smaller than on divergence and data on rare SNPs in protein coding regions are sparse. Thus, the analysis of allele frequency distribution may lack statistical power.

Our analysis suggests that mutation rates can be used in computational methods to predict which amino acid replacements are deleterious [32]: a replacement that occurred at a highly mutable site is more likely to be deleterious. Currently, prediction methods rely on the properties of an encoded amino acid sequence, its conservation between species, and the properties of the corresponding protein. Our analysis suggests that taking the DNA-level features of an amino acid replacement into account will increase the accuracy of prediction of its effect on protein function.

## Materials and Methods

To determine the impact of CpG context on mutation rates we constructed a human-chimpanzee-orangutan alignment for a ∼1 Mb piece of orangutan genomic sequence (gi:119380173), and analyzed it assuming parsimony. To study the impact of CpG context on the rate of evolution, we constructed human-chimpanzee-orangutan and human-chimpanzee-macaque alignments of coding regions of individual genes by finding the orthologous macaque gene for each UCSC human-chimpanzee pair with the by-directional best BLAST hits approach [33]. We also repeated the analysis on just two sequences assuming equilibrium CpG content (data not shown). This analysis resulted in similar estimates.

For the analysis of intrahuman diversity we used a comprehensive and systematic Applera dataset [27]. Chimpanzee nucleotides corresponding to human SNP positions were identified using the SNP UCSC genome track [34]. Applera set is gene centric. Therefore, for the analysis of non-coding diversity, we used randomly ascertained SNPs from the Perlegen set [35]. We also verified that coding SNPs from the Perlegen dataset produced estimates highly similar to those based on the Applera dataset. We analyzed each population separately and excluded SNPs, which were fixed in the population and could not be mapped to chimpanzee nucleotides (≈4.6%).

Statistical analysis was carried out using R statistical package v2.7.0 [36]. *p*-Values for individual amino acid residue contingency tables were computed by Monte Carlo simulations with the number of replicates B = 10^6^. To obtain combined *p*-values we used Stouffer's z-scores [37] and Fisher's sum of logs of *p* [38] methods. Cochran-Mantel-Haenszel test of conditional independence [39] was utilized to ensure there was no three-way interaction with the amino acid residue type.
