# Correction: Hypermutable Non-Synonymous Sites Are under Stronger Negative Selection

**DOI:** 10.1371/annotation/a81b1fab-890c-447b-a308-5bc8ca3eb21d

**Published:** 2008-12-10

**Authors:** Steffen Schmidt, Anna Gerasimova, Fyodor A. Kondrashov, Ivan A. Adzhubei, Alexey S. Kondrashov, Shamil Sunyaev

The fourth author's name was spelled incorrectly. The correct name is: Ivan A. Adzhubei. The correct citation is: Schmidt S, Gerasimova A, Kondrashov FA, Adzhubei IA, Kondrashov AS, et al. (2008) Hypermutable Non-Synonymous Sites Are under Stronger Negative Selection. PLoS Genet 4(11): e1000281. doi:10.1371/journal.pgen.1000281

